# Plant Triterpenoid Crosstalk: The Interaction of Brassinosteroids and Phytoecdysteroids in *Lepidium sativum*

**DOI:** 10.3390/plants9101325

**Published:** 2020-10-07

**Authors:** Danuše Tarkowská, Eliška Krampolová, Miroslav Strnad

**Affiliations:** Laboratory of Growth Regulators, Institute of Experimental Botany, Czech Academy of Sciences and Palacky University, CZ-78371 Olomouc, Czech Republic; E.Krampolova@seznam.cz (E.K.); miroslav.strnad@upol.cz (M.S.)

**Keywords:** triterpenoids, brassinosteroids, phytoecdysteroids, *Lepidium sativum*, plant growth

## Abstract

Plant steroid alcohols, plant sterols, are essential components of cell membranes that perform many functions. Their most prominent function is maintaining membrane semipermeability and regulating its fluidity through their specific interaction with phospholipids and membrane proteins. This work is focused on the study of the interaction of two groups of plant sterols, brassinosteroids (BRs) and phytoecdysteroids (PE). Steroid substances belonging to both groups are important signaling molecules essential for plant growth and development, but while the first group has all the known attributes of plant hormones, the second lacks hormonal function in plants. The aim of this preliminary study was to determine at what concentration level and to what extent substances of this type are able to interact with each other, and thus influence the early growth and development of a plant. It was found that exogenously applied PE 20-hydroxyecdysone (20E) significantly reduced the level of endogenous BRs in four-day-old garden cress (*Lepidium sativum*) seedlings. On the other hand, exogenously applied BRs, 24-*epi*brassinolide (*epi*BL), caused the opposite effect. Endogenous 20E was further detected at the picogram level in garden cress seedlings. Thus, this is the first report indicating that this plant species is PE-positive. The level of endogenous 20E in garden cress seedlings can be decreased by exogenous *epi*BL, but only at a relatively high concentration of 1·10^−6^ M in a culture medium. The image analysis of garden cress seedlings revealed that the length of shoot is affected neither by exogenous BRs nor PE, whereas the root length varies depending on the type and concentration of steroid applied.

## 1. Introduction

**Figure 1:**
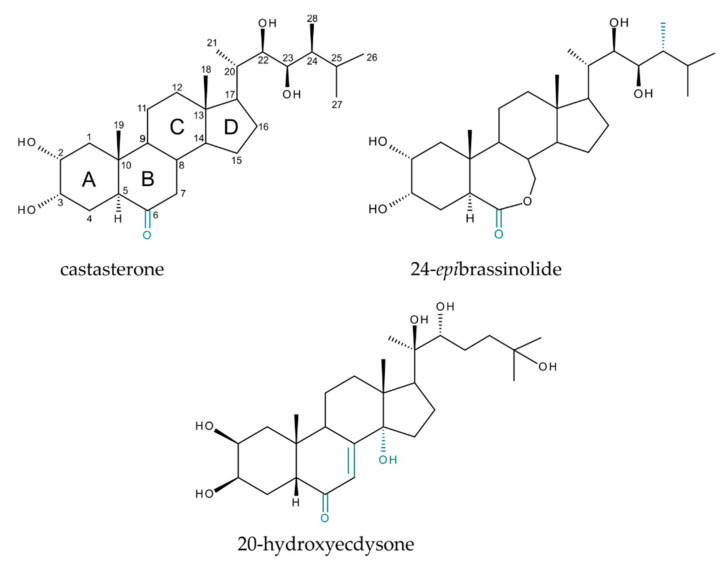
Chemical structure of prominent representatives of brassinosteroids (castasterone, 24-*epi*brassinolide) and phytoecdysteroids (20-hydroxyecdysone, 20E).

Plants, lacking motility, have never developed a nervous system, but they have evolved an intricate network of signaling molecules—chemical messengers—that play an indispensable role in the regulation of their responses to environmental stimuli, as well as their growth and development [1]. Brassinosteroids (BRs) and phytoecdysteroids (PEs) belong to a family of plant sterols that are a part of this network, and are thus active players in cell–cell communication in planta [2,3]. Chemically, both of these tetracyclic triterpenoid classes comprise of C_27_ to C_29_ polyhydroxylated steroid structures with an oxygenated B-ring (Figure 1, marked in teal). 

**Figure 2:**
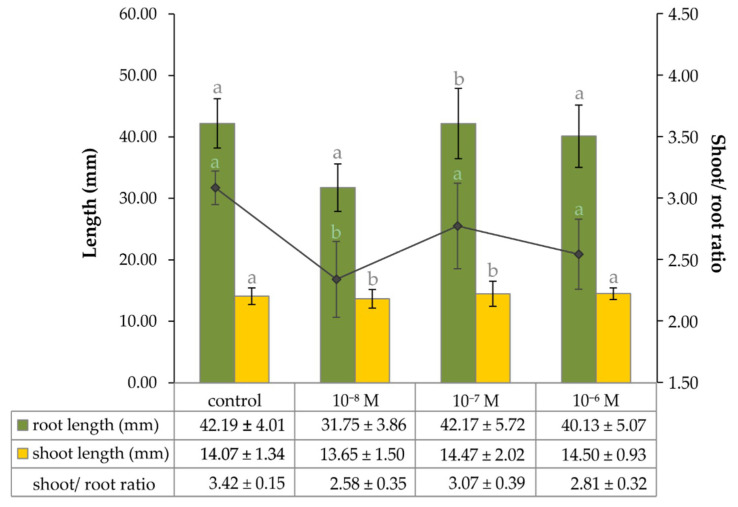
The graph of dependence of the shoot and root growth of four-day-old *Lepidium sativum* seedlings on the concentration of exogenous 20E. The data/error bars represent the mean/standard deviation of six independent measurements.

However, there are a few structural differences between these two groups of phytosterols that ultimately have a direct effect on the dissimilarity of their biological activity, namely: (1) the B-ring in BRs bears only a carbonyl group at C-6, while PEs have a characteristic 14α-hydroxy-7-en-6-one chromophoric moiety (Figure 1, marked in teal); (2) the orientation of hydroxyl groups at C-2, C-3, and C-22 is mirrored; and lastly, (3) the junction of the A- and B-rings is in a cis-orientation in the skeleton of PEs, while BRs have a trans-configuration. Importantly, six-membered B-ring in BRs may be expanded during their biosynthesis to form a lactone, which occurs in the case of brassinolide (BL) and its epimer 24-*epi*brassinolide (*epi*BL, Figure 2). These 7-oxalactone BRs show greater biological activity than 6-oxo types (e.g., castasterone, Figure 2) [4]. However, the structure of the side chain in PEs and BRs could be more important for their biological activity than that of their steroidal core skeleton, as demonstrated by Watanabe [5]. He found that hydroxylation at C-20 is detrimental to the plant’s hormonal activity of BRs, whereas the stereochemistry of the hydroxy group at C-22 is of pivotal importance for the molting hormonal activity.

BRs are generally accepted as plant hormones with known receptor [6] and well-described biological functions [7], while PEs are most often regarded as signaling molecules providing protection against non-adapted insects and/or soil nematodes [8,9] without hormonal properties. Whereas BR receptors show a high specificity, PEs exhibit weak or no activity in bioassays of BRs [10,11] and vice versa. Nevertheless, there are a range of physiological processes that are affected by both BRs and PEs, although the mechanism of their mutual interaction is not known yet. For instance, selected representatives of BRs (*epi*BL) and PEs (20E) have been shown to increase the yield of photosynthesis [12], and exogenously applied *epi*BL induces changes in PE content as well as PE profiles in the plant tissue [13].

The aim of this work was to show that the presence of exogenous PEs can also cause changes in the levels of endogenous steroid hormones, BRs. For this purpose, an earlier well-established sensitive analytical technique based on ultra-high-performance liquid chromatography coupled to tandem mass spectrometry (UHPLC–MS/MS) was used [14]. 

## 2. Results and Discussion

### 2.1. The Effect of Exogenous Phytoecdysteroid on Growth of Lepidium Seedlings and the Level of Endogenous Brassinosteroids

The data obtained by the image analysis of four-day-old garden cress seedlings showed that the length of their shoots was not affected by the presence of 20E at all, and reached an average of 14.2 mm in all of the experimental setups (Figure 2). The roots of the same seedlings were found to be about three times longer compared to corresponding shoots. A significant change occurred only when the growing media was treated with 20E at a concentration of 1·10^−8^ M, when the length of seedlings’ roots decreased by 23.5 %. Therefore, the trend of shoot-to-root ratio reflects the trend observed for the root length (Figure 2).

**Figure 3:**
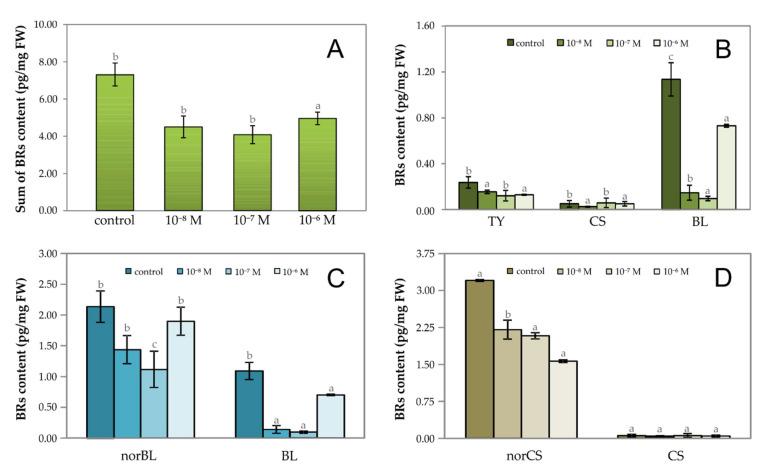
The effect of 20E at different concentrations on the (**A**) total content of endogenous brassinosteroids (BRs); (**B**) the content of typhasterol (TY), castasterone (CS), and brassinolide (BL); (**C**) the level of norBL (28-norbrassinolide) vs. BL; and (**D**) the level of norCS (28-norcastasterone) vs. CS in four-day-old seedlings of *Lepidium sativum* grown in vitro. Data/error bars represent the mean/standard deviation of three independent determinations.

By determining the levels of naturally occurring BRs in garden cress seedlings grown on media with 20E at the indicated concentrations, it was found that this PE generally reduced the levels of endogenous BRs by an average of 38% compared with the control plants (Figure 3A). The greatest influence for 20E was at a concentration of 1·10^−7^ M, where there was a reduction in the total level of BRs by 44% compared with the control, i.e., garden cress seedlings grown on a medium without 20E. This observation might be explained by the ability of PEs to affect BRs biosynthesis by so far unknown mechanisms. By analyzing the profile of BRs arising at the end of the early C-6 oxidation biosynthetic pathway (typhasterol (TY) → castasterone (CS) → brassinolide (BL)), we found that 20E had the greatest effect on the level of the most biologically active BRs, BL (Figure 3B), which is the end product of the biosynthetic pathway of all BRs [7]. Its analogue, lacking the CH_3_ group at carbon 28, 28-norbrassinolide (norBL), is the second most abundant BR of all endogenous BRs detected in the garden cress (accounting for 31.7 % of all BRs; Figure 3C) and the trend in its levels exactly imitated the situation described for the sum of all BRs in the studied tissue (Figure 3A). It also coincided with the trend in BL levels (Figure 3C). However, the highest level was determined for 28-norcastasterone (norCS; accounting for 43.5 % of all BRs), decreasing with increasing concentration of 20E in the culture medium, and reaching 68.8 % to 48.8 % of the norCS content in the control plants untreated with 20E (Figure 3D).

**Figure 4:**
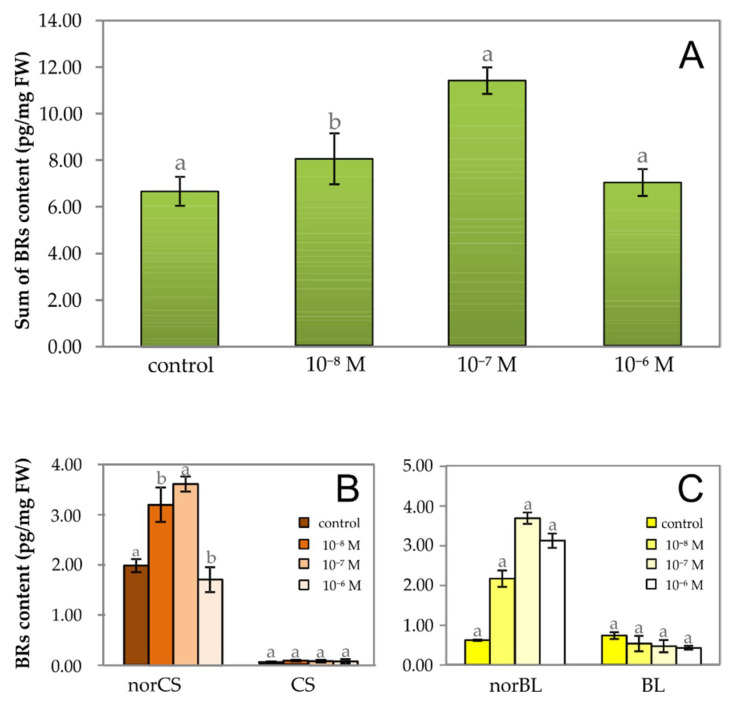
The effect of exogenous *epi*BL (24-*epi*brassinolide) at different concentrations on the (**A**) total content of endogenous BRs, (**B**) the level of norCS vs. CS, and (**C**) the level of norBL vs. BL in four-day-old seedlings of *Lepidium sativum* grown in vitro. Data/error bars represent mean/standard deviation of three independent determinations.

Reciprocally, the BRs levels were also quantified in garden cress seedlings cultured on one of the selected BRs (*epi*BL) at the same concentrations as in the case of 20E, i.e., at 1·10^−6^ M, 1·10^−7^ M, and 1·10^−8^ M in the medium. It was found that *epi*BL acts on the levels of endogenous BRs exactly the opposite way of the effect of 20E. *Epi*BL increased the levels of endogenous BRs by an average of 32 % (Figure 4A). It had the greatest influence on the levels of BRs at a concentration of 1·10^−7^ M, when the total amount of BRs in the garden cress tissue increased by up to 69.9 % compared with the control. This increase was 19.8% and 4.6% for 1·10^−8^ M and 1·10^−6^ M of *epi*BL in the medium, respectively. An analysis of the BR profiles revealed that among all of the detected BRs, the highest levels observed were again of norCS (Figure 4B), followed by norBL (Figure 4C). This is in agreement with the data obtained in the experiment with the cultivation of garden cress seedlings on a medium with 20E (Figure 3). The trend of norCS levels across exogenous concentrations of *epi*BL applied in garden cress culture media corresponded with the trend for the sum of all BRs (Figure 4B vs. Figure 4A). It follows from the above that the PEs and BRs in garden cress tissue have an antagonistic effect, and exogenously applied BRs have a much greater influence on the biosynthesis of BRs than PE at the same concentration (see below). This might be related to the presence of the BR receptors in plant cells, which transmitted an exogenous BR signal much more sensitively than that of PE under the same experimental conditions. The reports about antagonism between PEs and BRs are relatively limited. However, Lehmann et al. showed that BRs bind competitively to ecdysteroid (EC) receptors partially purified from larvae of the blowfly *Calliphora vicina* and inhibit biological responses to 20E [15]. Furthermore, using *Drosophila melanogaster* B_II_ cell bioassay, a series of synthetic hybrid BR/EC structures have been assessed for their EC agonist/antagonist activities where only three compounds weakly antagonized the action of 20E at 5⋅10^−8^ M [16]. Nevertheless, none of the ECs showed activity in the rice lamina inclination test, a well-established BR bioassay, demonstrating the high specificities of the insect EC receptor and the plant BR receptor.

### 2.2. The Effect of Exogenous Brassinosteroid on Growth of Lepidium Seedlings and the Level of Endogenous Phytoecdysteroid

**Figure 5:**
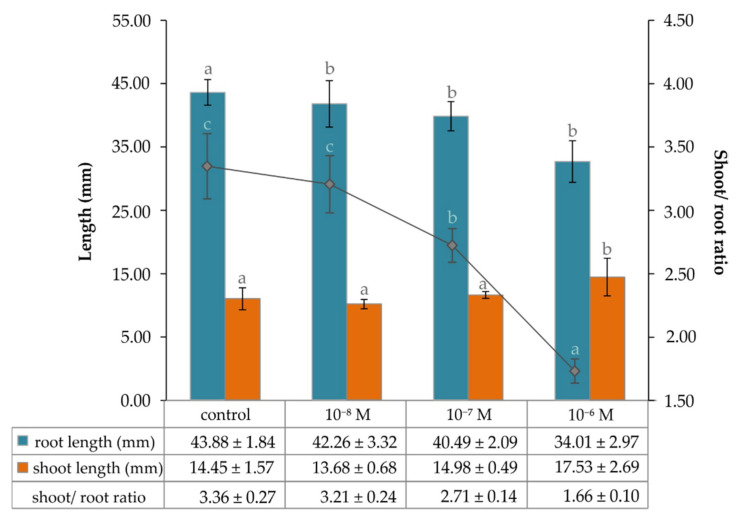
The graph of dependence of shoot and root growth of four-day-old *Lepidium sativum* seedlings on the concentration of exogenous *epi*BL. The data/error bars represent the mean/standard deviation of six independent measurements.

Based on the image analysis of four-day-old *Lepidium* seedlings grown on an agar MS (Murashige Skoog) medium containing either 1⋅10^−8^ M, 1⋅10^−7^ M, or 1⋅10^−8^ M *epi*BL, it was found that the length of their roots decreased in a concentration-dependent manner, while the length of the shoots seemed to increase only moderately (Figure 5). The root length of the control plants grown on media without *epi*BL was comparable with those grown on media containing 1⋅10^−8^ M of *epi*BL, which then slowly decreased with the increased concentration of these BRs in culture media, and reached about 77 % of the control root length at 1⋅10^−6^ M of *epi*BL. The length of the seedlings’ shoots was not affected for those grown in the presence of *epi*BL at concentrations of 1⋅10^−8^ M and 1⋅10^−7^ M, whereas the concentration of 1⋅10^−6^ M of this plant growth regulator in the culture medium caused an increase in this parameter by 21%. The shoot-to-root ratio therefore decreased by 49%, from 3.36 (control plants) to 1.66 (plants grown in the presence of 1⋅10^−6^ M *epi*BL; Figure 5).

**Figure 6:**
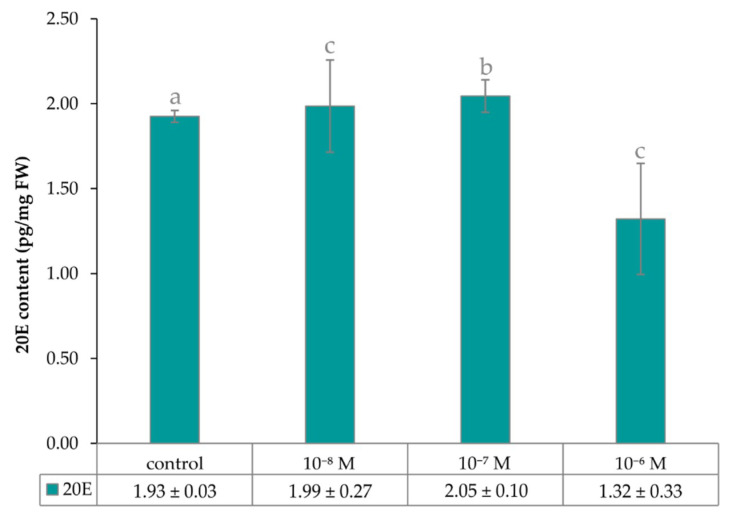
Graph of 20-hydroxyecdysone (20E) content in four-day-old seedlings of *Lepidium sativum* grown in vitro on media containing different concentrations of *epi*BL. Data/error bars represent the mean/standard deviation of three independent determinations.

The quantitative analysis of 20E in extracts of four-day-old seedlings of garden cress by UHPLC-MS/MS confirmed the earlier published finding [12] that exogenous *epi*BL is able to affect the endogenous level of PEs. As can be seen from Figure 6, the amount of 20E was about 1.9 pg/mg of fresh weight (FW) in the control *Lepidium* seedlings (no *epi*BL in growth medium), and remained almost unchanged even in the presence of 1·10^−7^ M and 1·10^−8^ M of *epi*BL. This substance, at a concentration of 1·10^−6^ M, caused the level of endogenous 20E to drop to 1.3 pg/mg FW, which is about 32%. This is in agreement with data of Kamlar et al. [13], who used *Spinacia oleracea* as a model plant, examining PEs for their experiment. In the case of garden cress, this is the first report of the presence of 20E in this plant species to our knowledge.

## 3. Material and Methods

### 3.1. Plant Material, Growing, and Cultivation Conditions

The seeds of *Lepidium sativum* L. (Moravoseed Plc., Czech Republic) were surface-sterilized using a 70% ethanol solution containing 0.1% of Tween^®^ 20, and were cultivated in vitro on vertical plates (120 × 120 × 17 mm; P-LAB, Czech Republic; 10 seeds in two separate lines/plate) containing 70 mL of Murashige Skoog (MS) basal growth medium including vitamins (4.4 g of MS salt/L; 3% sucrose; pH 5.6; agar 5.5 g/L—all purchased from Dutchefa Biochemie, the Netherlands). The cultivation was performed for four days under long day conditions of 18 h light, 23 °C/8 h dark, 18 °C, and light intensity 300 μmol⋅m^−2^⋅s^−1^ (Photon Systems Instruments Ltd., Czech Republic). After four days of cultivation, a picture of the seedlings was taken for image analysis (see below), and the seedlings were then collected, frozen in liquid nitrogen, and stored at −80 °C in a freezer until extraction and analysis.

In the experiment related to the study of BRs’ effect on PEs (20E), vertical plates containing 0 mol/L (control), 1⋅10^−6^ mol/L, 1⋅10^−7^ mol/L, and 1⋅10^−8^ mol/L of *epi*BL (OlChemIm Ltd., Olomouc, Czech Republic) in an agar MS medium were prepared, with three identical plates for each sample. Similarly, when studying the effect of 20E on the levels of BRs, vertical plates containing 0 mol/L, 1⋅10^−6^ mol/L, 1⋅10^−7^ mol/L, and 1⋅10^−8^ mol/L of 20E (Merck, Darmstadt, Germany) in an agar MS medium were each replicated three times.

The Java-based open source image-processing program ImageJ (the National Institutes of Health and the Laboratory for Optical and Computational Instrumentation, University of Wisconsin, USA) was used for image analysis of the garden cress seedlings. Deionized (Milli-Q) water obtained from a Simplicity^®^ UV Water Purification System (Darmstadt, Germany) was used to prepare all of the aqueous solutions. All of the other chemicals (analytical grade or higher purity) were from Biosolve Chimie (Dieuze, France).

### 3.2. Sample Preparation and Analysis of Brassinosteroids by Ultra-High-Performance Liquid Chromatography–Electrospray Tandem Mass Spectrometry (UHPLC–ESI–MS/MS)

The extraction and analysis of the BRs was performed in three technical replicates according to Tarkowska et al. [14], with some modifications. Briefly, fresh plant tissue samples of 10 mg fresh weight (FW) were homogenized to a fine consistency, with 1 mL of 60% acetonitrile (ACN) as the extraction solution. The samples were then extracted overnight after adding BRs internal standards (OlChemIm Ltd. Olomouc, Czech Republic). The crude extracts were centrifuged (36,670× *g*, 10 min, and 4 °C; Beckman Avanti™ 30), and the corresponding supernatants were further purified using polyamine solid phase extraction columns Discovery DPA^®^-6S (50 mg, Supelco^®^, Bellefonte, PA, USA). Finally, the samples were analyzed by UHPLC-MS/MS (Micromass, Manchester, UK). The quantitation of BRs was performed using the isotope dilution method [17] after processing the MS data with MassLynx software (version 4.2, Waters, Manchester, UK).

### 3.3. Sample Preparation and Analysis of Phytoecdysteroids by Ultra-High-Performance Liquid Chromatography–Electrospray Tandem Mass Spectrometry (UHPLC–ESI–MS/MS)

The extraction and analysis of PEs was performed according to Kamlar et al. [13], with some modifications. Fresh plant tissue samples of 10 mg FW were homogenized to a fine consistency with 1 mL of 80 % methanol (MeOH) as the extraction solution. The samples were then extracted overnight, followed by centrifugation of the crude extracts (36,670× *g*, 10 min, and 4 °C; Beckman Avanti™ 30) and purification of corresponding supernatants using polyamine solid phase extraction columns Discovery DPA^®^-6S (50 mg, Supelco^®^, Bellefonte, PA, USA). Finally, the samples were analyzed by UHPLC-MS/MS (Micromass, Manchester, UK).

### 3.4. Statistical Analysis

All data were subjected to a standard analysis of variance. The means were compared using Tukey’s HSD test, with the significance threshold set at 0.05. All calculations were performed using the Sigma Plot software v12.3. 

## 4. Conclusions

The experiments performed in this preliminary study show that the presence of 20E, the most widely distributed PE in nature, reduces the levels of endogenous BRs in four-day-old seedlings of *Lepidium sativum* belonging to the Brassicaceae family. The largest decrease in the sum of endogenous BRs was observed on the medium with 20E at a concentration of 1·10^−7^ M, while the greatest effect had the presence of exogenous 20E on the level of brassinolide, the most biologically active BR. The reciprocal treatment of culture media with exogenous *epi*BL caused the reduction in 20E endogenous level only at the highest concentration of 1·10^−6^ M used. The presence of endogenous PEs in this plant species has not yet been published, so this is the first report about *Lepidium sativum* being PE-positive.
